# Sports events as economic and political instruments: unpacking the dynamics of image creation through sport

**DOI:** 10.3389/fspor.2026.1784371

**Published:** 2026-06-29

**Authors:** Ilona Lejniece, Marina Kamenecka-Usova, Janis Židens, Signe Luika, Žanete Korde

**Affiliations:** 1 Faculty of Social Sciences, Riga Stradiņš University, Riga, Latvia; 2 Law Department, EKA University of Applied Sciences, Riga, Latvia; 3 RSU Latvian Academy of Sport Education, Riga Stradiņš University, Riga, Latvia; 4 Department of Health Psychology and Pedagogy, Riga Stradiņš University, Riga, Latvia

**Keywords:** image creation, soft power, sport governance, sport infrastructure, sports events, sportswashing

## Abstract

**Introduction:**

This article examines how sports events are conceptualized in scholarly literature as economic and political instruments of image creation, soft power projection, sport governance, reputational positioning, and sportswashing.

**Methods:**

The study adopts a systematic mapping/scoping review design using bibliometric performance analysis and science mapping to examine publications indexed in Scopus and Web of Science between 2015 and 2025.

**Results:**

The analysis identifies key thematic clusters, international collaboration patterns, and relationships between sport events, infrastructure development, governance, and political communication. The findings show that sportswashing appears as an embedded concept within a wider research landscape rather than as an isolated research stream. Contemporary image-building through sport is increasingly materialized through stadium construction, infrastructure megaprojects, and urban redevelopment.

**Discussion:**

Hosting global sporting events can enhance economic visibility and soft power, but it also raises ethical concerns, intensifies spatial inequalities, and produces uneven developmental outcomes. Identified research gaps include limited quantitative evidence on long-term economic effects, insufficient assessment of governance transparency, and underdeveloped comparative analysis of infrastructural legacies as political tools.

## 1 Introduction

In recent years, the intersection of sport, economics, politics, and international communication has attracted growing scholarly attention. Sports events, particularly major and mega-events, are increasingly analysed not only as entertainment spectacles but also as strategic instruments through which states, cities, organizations, and corporations pursue image creation, soft power projection, economic visibility, and political legitimacy. In this context, the soft power of sport can be understood as the ability of actors to achieve desired outcomes through attraction, visibility, and positive association generated by sport, rather than through coercion or financial incentives, while also serving broader policy objectives such as nation branding and diplomacy. Earlier studies on state soft-power strategies through sports mega-events similarly show how hosting can be used to pursue international visibility, symbolic capital, and diplomatic positioning (1). Within this broader field, sportswashing has emerged as a more specific and contested concept, referring to the use of investment in sport, event hosting, sponsorship, or sports infrastructure to offset reputational concerns in other political, social, or human-rights domains. This article, therefore, approaches sportswashing not as the sole explanatory frame, but as one concept embedded within wider debates on image creation, the soft power of sport, governance, and reputational politics (2–10).

A key dimension of sport-based image creation and sportswashing, further elaborated in Section 5.3 of this article, concerns the role of sports facilities and urban development as material mechanisms for the formation of reputational and political narratives. Stadiums, arenas, fan zones, transport corridors, and broader urban renewal projects form the physical infrastructure through which states embody political narratives of modernization, capability, and global integration (3, 8, 11). These infrastructural megaprojects (12) allow governments to signal progress, attract investment, and consolidate legitimacy while masking social or political tensions. As shown in recent scholarship, the built environment has become an increasingly central arena for sportswashing, linking symbolic communication to spatial and economic transformation.

This article aims to map and interpret the international scholarly literature on sports events as economic and political instruments of image creation, soft power projection, sport governance, and reputational politics. Within this wider framework, sportswashing is examined as a specific concept included in the Scopus and Web of Science search strategy and analysed through keyword co-occurrence mapping. Specifically, the study identifies dominant thematic clusters, maps international collaboration patterns, examines the relationship between sports events, infrastructure development, economic visibility, and political legitimacy, and highlights conceptual and methodological gaps in the field.

In this article, “mega sport events” refer to globally visible, large-scale events with extensive international media, infrastructural, economic, and political implications, such as the Olympic Games or FIFA World Cup, whereas “major sport events” refer to significant international or regional competitions that may be smaller in scale but still generate economic, political, and reputational effects.

The following research question guides the review: How has the scholarly literature from 2015 to 2025 conceptualized sports events as economic and political instruments of image creation, soft power, governance, and sportswashing?

To address this question, the review asks:

1) What thematic clusters structure the literature on sports events, image creation, soft power, governance, and sportswashing?
2) How does sportswashing appear within the co-word network relative to broader concepts such as sport governance, soft power, infrastructure, and economic analysis?
3) What geographical patterns characterize international research collaboration in this field?
4) What conceptual and methodological gaps remain for future research?

## 2 Methods

This article is designed as a systematic mapping/scoping review supported by bibliometric performance analysis and science mapping. This design is appropriate because the aim is not to synthesize intervention effects or estimate pooled outcomes, but to map the structure, thematic organization, and geographical distribution of scholarly literature on sports events as economic and political instruments. The review combines quantitative bibliometric indicators with qualitative interpretation of thematic clusters to identify how concepts such as image creation, soft power, sport governance, infrastructure development, and sportswashing are positioned within the field.

Bibliometric analysis applies three types of indicators: quantitative, for measuring productivity; qualitative, for evaluating research impact; and structural, for analysing interrelationships among publications and their components (13). Two principal methodological approaches were used: performance analysis, focusing on productivity and impact, and science mapping, which explores the relationships between publications (14). The methodological design and data parameters of this bibliometric analysis are summarized in Table 1, adapted from Öztürk et al. (15).

**Table 1:** Overview of bibliometric methods and data characteristics used in the study.

Component	Description
Database source (1)	Riga Stradiņš University Library Portal (Scopus Elsevier Online)
Search strategy (queries)	TITLE-ABS-KEY (sport* event*) AND TITLE-ABS-KEY (sportswashing*) AND TITLE-ABS-KEY (economic* aspect* analysis)—no documents. TITLE-ABS-KEY (sport* event*) OR TITLE-ABS-KEY (sportswashing*) AND TITLE-ABS-KEY (economic* aspect* analysis)—no documents. TITLE-ABS-KEY (sport* event*) OR TITLE-ABS-KEY (sportswashing)—77 documents. TITLE-ABS-KEY (sport* event*) AND TITLE-ABS-KEY (sportswashing)—no documents. TITLE-ABS-KEY (sport* event*) AND TITLE-ABS-KEY (sportswashing) OR TITLE-ABS-KEY (economic* aspect)—no documents. TITLE-ABS-KEY (sport* event) OR TITLE-ABS-KEY (sportswashing) OR TITLE-ABS-KEY (economic* aspect analysis)—1,226 documents. Rationale for final query: selected to retain sportswashing as an explicit search term while capturing the broader literature on sports events as economic and political instruments; narrower queries produced no or insufficient records for robust bibliometric mapping.
Screening and exclusion logic	Metadata-level screening based on title, abstract, author keywords, publication year, document type, subject area, and source information. Records outside 2015–2025, non-article/review formats, records lacking usable bibliographic metadata, and thematically non-relevant terms identified during keyword cleaning were excluded from thematic mapping.
Time span	2015–2025
Subject areas included	Social Sciences; Business, Management and Accounting
Document types	1,172 articles; 54 reviews
Total documents	1,226
Journals represented	536
Total authors	5,428
Average authors per document	8.32
International co-authorship rate	23%
Average publication age	4.51 years
Total author keywords	6,012
Keyword cleaning procedures	Synonym merging and semantic consolidation using a validated thesaurus. Examples include: sport event/sports event/sporting event → sport event; mega event/mega-event/mega sport event → mega sport event; major event/major sport event/major sporting event → major sport event; soft-power/soft power strategy/soft power projection → soft power; Generic or non-informative terms such as study, research, paper, approach, method, analysis, impact, effect, model, and review were removed.
Data cleaning process	Manual normalization in Microsoft Excel; thesaurus validated by two independent researchers; secondary refinement in VOSviewer. The term sportswashing, including variants such as sport washing and sports-washing, was retained as a distinct concept because it was central to the review question and explicitly included in the Scopus and WoS search strategies. Infrastructure-related terms such as stadium, stadiums, sport facility, and sports facilities were harmonized where conceptually equivalent. Non-relevant terms outside the review focus, including tobacco, alcohol, doping, medical/health, nutrition, age, animals, gender, racism, and sexism, were excluded during secondary refinement when not directly linked to sport events, image creation, governance, or sportswashing.
Software used	VOSviewer (v.1.6.19); Biblioshiny
Analytical techniques	Performance analysis; co-word analysis; co-authorship network analysis; inductive content analysis
Threshold criteria	≥7 keyword occurrences; ≥11 joint publications for country-level mapping Rationale: thresholds selected to balance network interpretability and inclusion of emerging but conceptually relevant terms, including sportswashing
Languages	All languages included (dominant: English) No language restrictions were applied during database searching. All languages were eligible for inclusion; however, the final corpus was dominated by English-language publications due to the indexing profile of Scopus and WoS. Non-English records were retained when titles, abstracts, keywords, or database metadata were available in English or could be reliably interpreted.
Database/Indexes	Scopus (Elsevier)
Affiliations	Global academic and research institutions
Countries/regions	All
Output visualisations	Co-occurrence networks; density maps; cluster visualisations
Database source (2)	Riga Stradiņš University Library Portal (Web of Science Core Collection Online)
Search strategy (queries)	TS = (“sport* event*” OR “sportswash*” OR “economic*” “aspect*” analys*)
Time span	2015–2025
Subject areas included	Sport Sciences WoS Sport Sciences was used as a broader validation field because WoS subject categories do not map directly onto Scopus subject areas. The comparison therefore relied on harmonized thematic subsets rather than identical disciplinary categories.
Document types	Articles; Reviews
Total documents	5,296
Multi-database query validation	Scopus (Elsevier) and Web of Science (WoS)
Databases/initial exports	Scopus (*N* = 1,226); WoS (*N* = 5,296) WoS export is broader and includes extensive coverage of sports medicine/physiology; therefore, direct comparison is conducted on harmonized, thematically equivalent subsets.
Thematically harmonized subset (same inclusion logic in both databases)	2015–2025; Document types: Article/Review; query terms applied to title/abstract/keywords fields. Scopus (*n* = 535) WoS (*n* = 468)
DOI coverage (multi-database robustness indicator)	Scopus: 535 records; DOI available for 502 unique records (33 without DOI); WoS: 468 records; DOI available for 441 unique records (27 without DOI).
Metadata matching to the common DOI set	Publication year match: 97.2% (minor discrepancies likely due to early access/indexing differences) Title similarity (normalized string similarity): mean 0.979; 94.4% of cases ≥ 0.90.
Thematic structure (macro level; keyword co-occurrence network):	Scopus: 3 clusters; WoS: 4 clusters (WoS more frequently separates reputation/communication subtopics); Conclusion: cluster counts may differ, but the core thematic structure and hierarchical organization are reproducible across databases.
Conceptual interlinks (cross-links between clusters)	Conceptual interconnections are reproducible across both databases; differences reflect granularity rather than underlying linkage logic.
Conceptual coherence of “sportswashing”	Scopus: bridging concept between event governance and political legitimacy/reputation; WoS: primarily within politics/ethics, but direcly connected to sports events/mega-events node; Conclusion: “sportswashing” appears as an integrated, embedded concept rather than an isolated research stream.
Methodological differences between databases	Scopus more often merges governance and political sub-nodes; WoS more often separates reputation/sponsorship topics; Differences are expected due to coverage and indexing; the key result is reproducibility of core structure and link architecture.
Software used	VOSviewer; Excel; Biblioshiny

**Figure 1:**
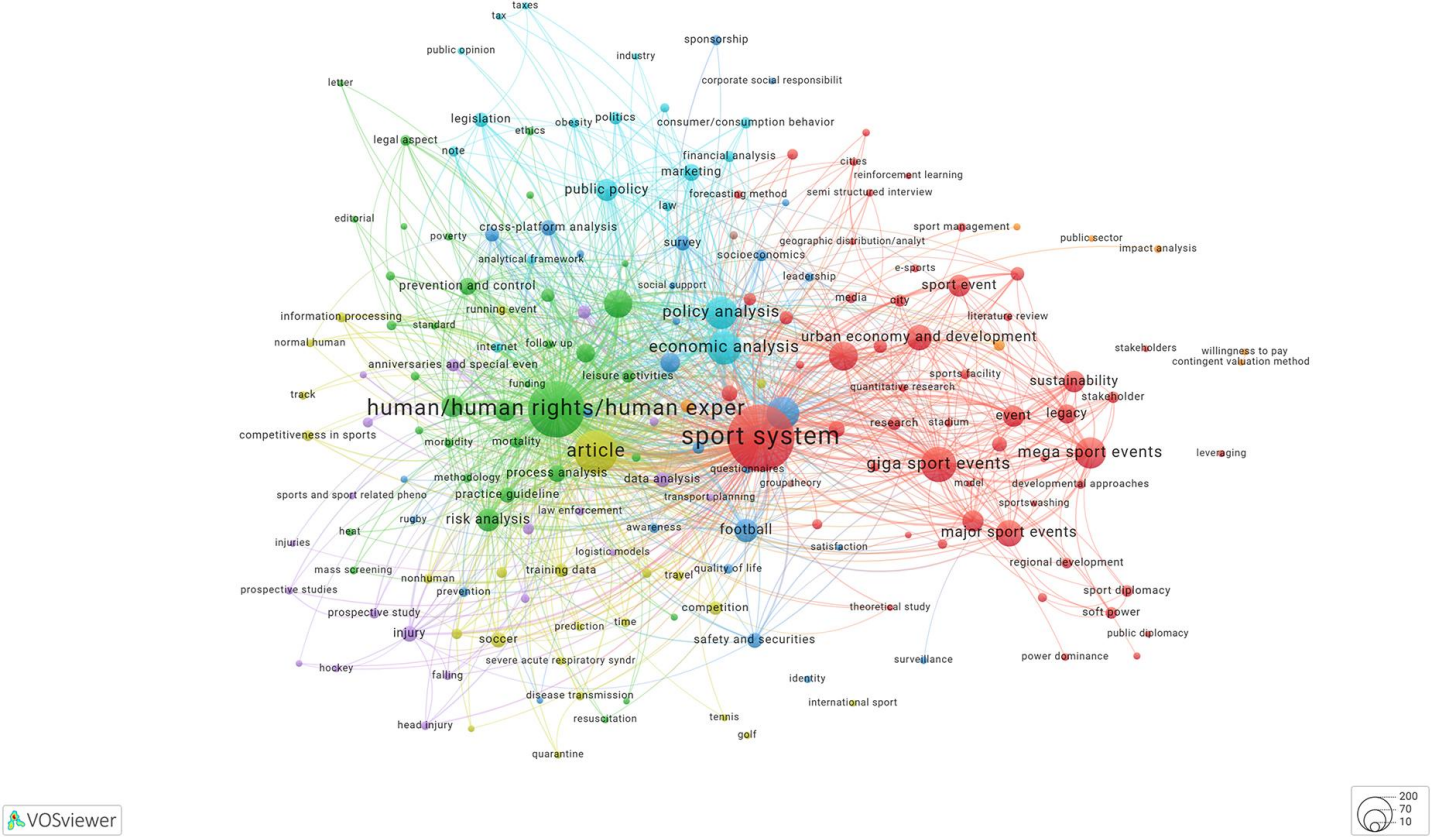
Thematic co-word clusters in sport event research (2015–2025). Network visualization generated using VOSviewer based on Scopus data (2015–2025). The figure displays eight thematic clusters and highlights the structural centrality of the red, blue, and green clusters within the field.

**Figure 2:**
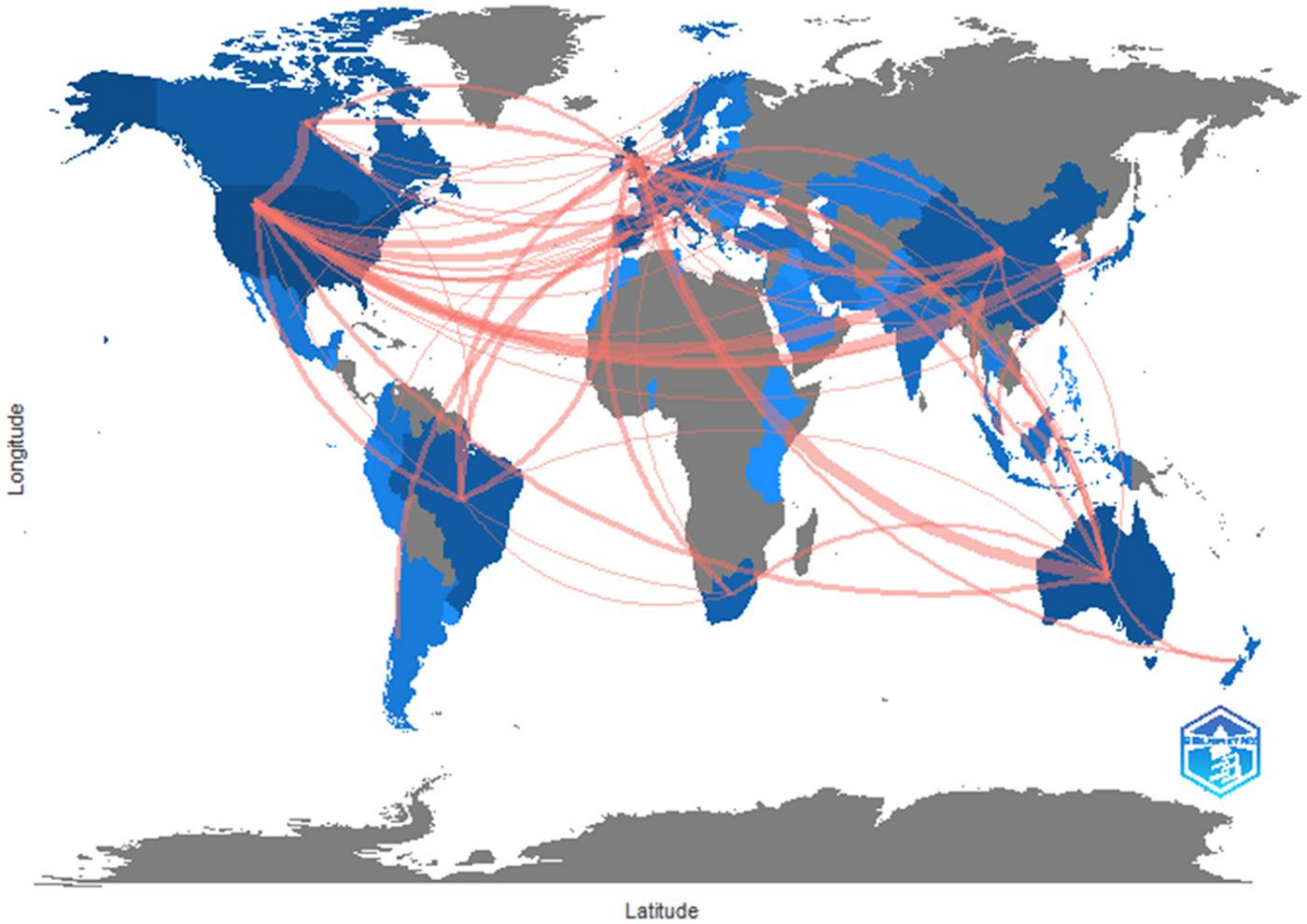
Global co-authorship collaboration network in sport event research (2015–2025). Collaboration map generated using Biblioshiny based on Scopus co-authorship data (2015–2025). The figure illustrates international collaboration flows across Europe, the Americas, and the Asia-Pacific region, highlighting regional asymmetries and transregional connectivity.

Summary of methodological parameters, including database coverage, search strategy, inclusion and exclusion criteria, keyword cleaning procedures, analytical tools (VOSviewer, Biblioshiny, Excel), and multi-database data validation against the Web of Science (WoS). The table documents all data-processing steps that form the empirical basis for Figures 1, 2.

Secondary bibliometric data were retrieved from the Scopus database (Elsevier), covering peer-reviewed publications from 2015 to 2025 in the subject areas of Social Sciences and Business, Management, and Accounting. Data were collected via the Riga Stradiņš University online library portal on September 1, 2025. The final search query-TITLE-ABS-KEY (“sport* event” OR “sportswashing” OR “economic* aspect analysis”)-was the sixth iteration of a systematically refined strategy, as the previous five queries produced either no results or an insufficient corpus for robust analysis. The query returned 1,226 records, comprising publications that contained at least one of the specified terms (including wildcard variants) in the title, abstract, or author keywords.

Eligible records were limited to published articles and reviews indexed in Scopus between 2015 and 2025. Records were excluded if they fell outside the selected time period, were not classified as articles or reviews, lacked usable bibliographic metadata, or were thematically unrelated after keyword and metadata screening. The same inclusion logic was applied to the Web of Science cross-check, while recognizing that database coverage, indexing practices, and disciplinary classifications differ between Scopus and WoS.

The iterative query refinement process is reported in Table 1 to ensure transparency. The first five queries were tested to assess whether narrow Boolean combinations of “sport event”, “sportswashing”, and “economic aspect analysis” would produce a sufficiently focused but analysable corpus. Several of these queries produced no documents, while one narrower query returned only 77 records. The sixth query was selected because it provided a broader corpus suitable for science mapping while retaining sportswashing as an explicit search term. This was important because one objective of the review was to determine whether sportswashing appears as an embedded concept within the wider literature on sport events, image creation, soft power, and economic-political instruments.

To meet multi-database evidence expectations for bibliometric research, the Scopus-based corpus was validated through an independent query cross-check against the Web of Science (WoS) Core Collection. WoS records were exported separately and filtered using the same inclusion logic as in Scopus (publication years 2015–2025; document types Article/Review; query terms applied to the title, abstract, and keyword fields). A harmonized, thematically equivalent subset was constructed in both databases to enable a like-for-like comparison (see Table 1).

The WoS cross-check was not used to replace the Scopus dataset but to assess the robustness of the thematic structure across databases. Because Scopus and WoS differ in journal coverage, indexing practices, and disciplinary emphasis, the comparison focused on harmonized and thematically equivalent subsets rather than identical record counts. This procedure helped reduce database-specific bias and supported the interpretation of sportswashing as a concept connected to, rather than isolated from, broader themes of sport governance, reputation, and political communication.

Keyword data were cleaned and standardized in Microsoft Excel by merging synonymous and semantically similar terms and eliminating redundant or overly generic keywords. For example, variants such as “sport event”, “sports event”, and “sporting event” were consolidated; related forms of “mega event” and “mega sport event” were standardized; and terms such as “soft-power” and “soft power strategy” were harmonized under “soft power”. The term “sportswashing” was retained as a distinct keyword because it represents a specific and contested concept within the broader image-creation and soft-power literature, and it was explicitly included in the Scopus and WoS search strategies. The resulting thesaurus was independently reviewed and validated by two researchers before integration into the analysis. Additional refinement was conducted in VOSviewer by removing non-relevant thematic keywords to ensure conceptual alignment. A structured overview of the data cleaning workflow is presented in Table 1.

For each record, bibliographic metadata were exported, including title, abstract, author keywords, source title, publication year, document type, authors, affiliations, country information, citations, DOI, and indexed subject area. These metadata formed the basis for performance analysis, co-word analysis, co-authorship mapping, and descriptive trend analysis.

Screening was conducted at the metadata level because the study is a bibliometric mapping review rather than a full-text evidence synthesis. Titles, abstracts, author keywords, and source information were inspected to identify records relevant to sport events, sport governance, image creation, soft power, infrastructure, and sportswashing. Records were retained in the bibliometric corpus if they met the database, year, document type, and subject area criteria; thematic refinement was subsequently performed through keyword cleaning, thesaurus validation, and VOSviewer-based exclusion of non-relevant terms.

Keywords appearing at least seven times and countries with eleven or more joint publications were included in the mapping process. These thresholds were selected to balance inclusiveness and interpretability: lower thresholds produced overly fragmented maps with many weakly connected terms, while higher thresholds risked excluding emerging but conceptually important terms, including sportswashing. Retaining the seven-occurrence keyword threshold allowed sportswashing to remain visible in the co-word network and enabled the study to assess its position relative to broader concepts such as soft power, sport governance, economic analysis, and urban development.

Bibliometric mapping was conducted using VOSviewer and Biblioshiny (16), both well-established tools for science mapping and co-occurrence analysis (17).

Network visualization illustrated nodes representing authors, keywords, or countries, with node size indicating publication or citation frequency and links representing collaborative or thematic connections (17).

Descriptive statistics summarized publication trends and collaboration intensity, while inductive content analysis was applied to interpret the meanings and interrelations of keywords and co-authorship networks (18, 19). This mixed-method approach offered both quantitative mapping and qualitative interpretation, producing a systematic overview of how sporting events are conceptualized as instruments of economic and political influence.

Literature analysis was used to contextualize bibliometric patterns and identify dominant thematic streams within the field. The bibliometric corpus consisted of published peer-reviewed articles and reviews. Additional published policy-oriented and theoretical sources were used only for contextual interpretation, not as unpublished primary data. No language restrictions were applied during database searching. However, the corpus was dominated by English-language publications, reflecting the indexing profile of Scopus and WoS in this research area. Non-English records were retained when bibliographic metadata, titles, abstracts, and keywords were available in English or could be reliably interpreted through database-provided metadata. This approach reduced language-exclusion bias while recognizing that English-language scholarship remains structurally dominant in indexed sport-event research. Contextual interpretation considered high-impact journals and leading authors in globalization, sport sociology, and political communication, while the bibliometric corpus itself was determined by the predefined database, year, document-type, subject-area, and search-query criteria.

## 3 Quality appraisal and risk of bias assessment

A formal risk-of-bias assessment designed for intervention or outcome-based systematic reviews was not applicable because this study does not synthesize effect sizes, clinical outcomes, or causal effects. Instead, the review analyses bibliographic metadata and thematic structures. Methodological transparency and bias mitigation were addressed through clearly defined database searches, query refinement, Scopus-WoS cross-checking, keyword cleaning validation by two researchers, threshold reporting, and explicit acknowledgement of database, language, and keyword-search limitations.

## 4 Results

### 4.1 Cluster interactions and quantitative analysis of the three major clusters

The co-word analysis identified eight thematic clusters representing the conceptual landscape of research on sports events and related domains (see Figure 1)^1^.

Among these, three clusters—red, blue, and green—demonstrated the highest density, centrality, and total link strength, forming the structural core of the network.

The red cluster is the largest and most interconnected thematic component, comprising approximately 40%–45% of all nodes. It integrates concepts such as *sport system, sport event, event legacy, urban economy and development, sustainability, sportswashing, soft power,* and *sport diplomacy*. This cluster shows the highest total link strength and occupies the central map position, indicating its function as the primary conceptual anchor linking political, economic, and symbolic aspects of sport events. Its inter-cluster connectivity is strongest with the blue cluster (50%–60%) and moderate with the green cluster (25%–30%).

The blue cluster, representing 30%–35% of nodes, focuses on *public policy, economic analysis, governance, law*, and *marketing*. Its intermediate position and strong ties with the red cluster (50%–60%) highlight its bridging role between event-centred political-economic analysis and issues of institutional regulation. Connections to the green cluster are limited (15%–20%), positioning the blue cluster as the governance and policy backbone of the field.

The green cluster accounts for 20%–25% of nodes and includes *risk analysis, injury, prevention and control, morbidity,* and *training data*. Although more peripheral, it displays strong internal cohesion and maintains consistent links to the red cluster (25%–30%). Its ties to the blue cluster (10%–15%) are weaker, reflecting its indirect but relevant contribution to legitimacy and safety considerations surrounding sports events.

Smaller clusters capture emerging themes, including *e-sports*, *technological applications*, and sport-specific research areas (*football*, *tennis*, *soccer*), functioning as peripheral but expanding subfields within the network.

Keyword-level metrics (see Table 2) reinforce this hierarchy.

**Table 2:** Quantitative metrics of key thematic keywords across dominant bibliometric clusters, including overall co-word network characteristics and access to an interactive VOSviewer map.

Items	Cluster (color, number)	Links	Total link strength	Occurrences
Sport event	Red (1)	75	211	76
Mega sport event	Red (1)	91	454	153
Major sport event	Red (1)	94	379	111
Giga sport event	Red (1)	75	211	76
Sport system	Red (1)	184	2,939	705
Sportswashing	Red (1)	19	29	7
Sport diplomacy	Red (1)	19	42	21
Soft power	Red (1)	23	62	22
Governmentality	Red (1)	130	629	131
Local economy	Red (1)	55	150	29
Urban economy and development	Red (1)	106	409	103
Economic analysis	Blue (6)	163	988	177

Keyword-level bibliometric indicators, including occurrence frequency, link strength, and connectivity metrics across the major thematic clusters. The table reflects the hierarchical and structural roles of core concepts such as *sport system*, *sportswashing*, *soft power*, *economic analysis*, and *event legacy*. The inclusion of sportswashing at the seven-occurrence threshold allows the study to examine its position as an emerging and embedded concept within the broader co-word network.

*The sport system* exhibits the highest connectivity (184 links; total link strength 2939), confirming its central structural role. Event-related terms, including mega sport events, major sport events, and urban economy and development, demonstrate strong co-occurrence patterns, while economic connectors, such as economic analysis, form key bridging points across clusters. Political-symbolic keywords- *sportswashing, soft power, sport diplomacy*—appear at lower frequencies but show concentrated linkages indicative of a coherent subdomain focused on image, legitimacy, and international communication.

Although sportswashing appears with a lower occurrence frequency than broader terms such as sport system or economic analysis, its presence in the network is analytically significant because it meets the inclusion threshold and forms linkages with politically and reputationally relevant concepts. This supports the interpretation of sportswashing as an emerging and embedded sub-concept within the wider literature on sport events, image creation, soft power, and governance rather than as a separate or fully autonomous research stream.

Overall, the combined structural and quantitative evidence indicates a multilayered thematic architecture in which core systemic and event-related concepts define the network, governance themes provide institutional structure, and health-risk themes add contextual depth. Together, these clusters illustrate the interdependence of political, economic, and governance mechanisms in shaping contemporary research on sports events.

The structural role of these keywords also extends to patterns of international research cooperation, which are further examined in Section 4.2.

### 4.2 International collaboration patterns in global sport event research

The analysis of international collaboration patterns reveals a distinctly regionalized structure within global sport event research, shaped primarily by three major geographical blocs: Europe, the Americas, and the Asia-Pacific region. These regions form the core of the international co-authorship network, with collaboration intensity reflecting differentiated pathways of knowledge production and exchange across the global research system. The intensity of cross-regional interactions varies considerably, indicating differentiated pathways through which knowledge is produced, exchanged, and diffused across the global research landscape (see Figure 2).

Europe emerges as the most interconnected and structurally central region, exhibiting the highest density of both intra- and interregional collaborative ties. Strong internal cohesion- illustrated by recurrent partnerships among the United Kingdom, Germany, Spain, France, and the Nordic countries- reflects long-standing institutional linkages and aligned research agendas in sport governance and event management. Europe's extensive cross-regional engagement, particularly with the Asia–Pacific region (e.g., UK-China, Germany-Korea, Spain-China) and the Americas (e.g., UK-USA, Spain-Brazil, Germany-Canada), positions it as a global brokerage hub mediating scientific exchange across diverse research cultures.

The Americas display a collaboration pattern dominated by the outward-oriented profile of the United States, which maintains high-frequency partnerships with both European and Asia-Pacific countries on topics such as mega-event commercialization, sport policy, and political communication. Brazil represents the principal Latin American actor, maintaining collaborative ties with Portugal, Spain, France, and Germany, reflecting linguistic proximity and shared scholarly interest in Olympic and World Cup legacies.

The Asia-Pacific region demonstrates rapid expansion and increasing integration into global collaboration circuits. Countries such as China, Australia, Japan, Korea, Singapore, and Malaysia form a cohesive internal network, complemented by strong cross-regional ties with the United States and European partners. This expansion aligns with the region's growing academic capacity and thematic engagement with globalization processes, soft power strategies, sport diplomacy, and the political economy of mega-events.

Across all regions, collaboration patterns appear shaped by a combination of historical and linguistic ties, shared mega-event hosting experiences, policy-transfer networks, and concentrated research capacity in the US, UK, China, and leading European economies. Collectively, these dynamics indicate a trilateral structure in which Europe and the United States act as central knowledge exporters, while the Asia–Pacific region functions as a rapidly strengthening scientific hub increasingly embedded in global research flows.

### 4.3 Quantitative regional collaboration patterns and the structural dynamics of international research networks

The co-authorship network revealed a clearly regionalized structure dominated by Europe, the Americas, and the Asia-Pacific region (see Figure 2).

Europe accounted for the highest share of collaboration pairs (48.1%, *n* = 167) and demonstrated strong internal cohesion as well as extensive cross-regional partnerships with the Americas and Asia–Pacific. The Americas represented 34.6% (*n* = 120) of collaborations, with the United States forming the majority of high-frequency ties both within and outside the region. The Asia-Pacific region accounted for 29.4% (*n* = 102) and showed rapid growth and integration, with strong intra-regional ties and substantial collaboration with the United States and European partners.

Interregional collaborations represented 56.8% of all recorded ties, with the most active axis being Europe-Asia-Pacific (22.5%, *n* = 78), followed by Europe-Americas (18.7%, *n* = 65) and Americas-Asia-Pacific (15.6%, *n* = 54).

Within-region collaboration patterns were uneven:

- Europe: 29.4% (*n* = 102)
- Asia-Pacific: 16.4% (*n* = 57)
- Americas: 10.4% (*n* = 36)

Proportional weighting indicates that Europe holds the highest interregional centrality (41.2%), the Americas follow (33.8%), and the Asia–Pacific region (25.0%) is an emerging, increasingly influential hub.

Taken together, these results demonstrate that global sport event research is shaped by quantitatively uneven but functionally interdependent collaboration flows. Europe and the United States serve as dominant central nodes, while the Asia-Pacific region acts as a rapidly expanding integrator- collectively forming a trilateral knowledge architecture that mirrors geopolitical, economic, and cultural power distributions within global sport governance.

These geographical collaboration patterns are central to the article's contribution because they show how knowledge production on sports events as economic and political instruments is unevenly distributed across global regions. The prominence of Europe, the Americas, and the Asia-Pacific region indicates that debates on image creation, soft power, governance, and sportswashing are shaped not only by event-hosting practices but also by asymmetries in international research capacity and collaboration networks.

### 4.4 Annual trends and influencing factors shaping the transformation of research productivity

A longitudinal assessment of publication output revealed two distinct phases:

- Expansion phase (2015–2021)
- Annual publications increased from 70 (2015) to 151 (2021), with pronounced surges in 2018, 2020, and 2021. The overall trend reflects a sustained rise in research activity, culminating in the highest annual output recorded in the dataset.
- Recalibration phase (2022–2025)
- Publications declined by more than 20% in 2022, followed by stabilization during 2023–2025, with annual values ranging between 111 and 132. Despite this contraction, output levels remained above pre-2018 baselines, indicating structurally elevated research capacity.

Descriptive statistics confirm these patterns: the mean annual output for 2015–2025 is 113 publications, and the median is 111, reflecting concentration around a level significantly higher than that of the early period.

Publication dynamics between 2015 and 2025 were shaped by a combination of structural developments within the global research system, major international sports mega-events, geopolitical disruptions, public-health crises, and shifts in media and communication environments. From 2015 to 2021, output increased steadily due to expanded funding for sustainability and data-driven research, growing cross-disciplinary collaboration, and enhanced digital research infrastructures. Periodic spikes in productivity aligned with high-profile sports mega-events, including the 2016 Rio Olympics, the 2018 PyeongChang Winter Olympics, and the 2018 FIFA World Cup, which stimulated short-term thematic engagement in sport governance, performance analytics, soft power, and sportswashing.

The COVID-19 pandemic produced an exceptional surge in publications in 2020–2021, driven by emergency funding, accelerated peer-review processes, and intense scholarly focus on mass-gathering risk, public health, and the pandemic's disruption of global sport (20).

The postponement of the Tokyo 2020 Olympics and the implementation of biosecure sport environments created additional research pathways. A marked decline followed in 2022, reflecting the end of crisis-driven funding, institutional normalization, and the broader impact of geopolitical instability, particularly Russia's invasion of Ukraine, which reshaped international collaboration patterns and intensified research on sport diplomacy, authoritarian governance, and reputational politics.

From 2023 to 2025, publication volumes stabilized at levels above the pre-2018 baseline, influenced by shifting funding priorities, reduced pandemic urgency, and ongoing geopolitical realignments. Mega-events such as the 2022 Beijing Winter Olympics, the 2022 Qatar World Cup, and the 2024 Paris Olympics continued to catalyse research on sustainability, human rights, soft power, and sportswashing. Concurrently, transformations in global media ecosystems-especially the rise of digital and transnational communication- fuelled scholarly attention to framing, public diplomacy, and the political uses of sport.

Together, these factors demonstrate that publication trends were shaped by the interaction of long-term structural growth, crisis-induced accelerations, and recurrent thematic stimuli generated by sport mega-events, geopolitical shifts, and evolving global communication landscapes.

## 5 Discussion

### 5.1 General discussion- interpretation of bibliometric findings

The bibliometric analysis reveals that research on sport events as economic and political instruments has developed into a multidimensional field at the intersection of sport governance, political communication, image creation, soft power, and global economic strategy. Within this broader field, sportswashing appears as a specific and contested concept linked to legitimacy, reputation management, and political narrative formation. The prominence of the economic-governance cluster confirms that legitimacy, soft power, institutional behaviour, and resource allocation form the conceptual core of the field, supporting earlier arguments by Grix and Brannagan (3), Horne (6), and Boykoff (2). Keyword co-occurrence patterns show that sportswashing is understood not as an isolated tactic but as a structural mechanism embedded within broader forms of political influence and international image management.

The growing presence of themes associated with media, perception, and public diplomacy indicates that contemporary scholarship increasingly situates sportswashing within communicative and discursive processes. This trend aligns with empirical work by Gläßel, Scharpf, and Pearce (21), who demonstrate that public attitudes toward authoritarian mega-event hosts are significantly shaped by event framing and national media environment. The presence of terms linked to soft power, digital communication, and global reputation reflects a shift from event-centred analysis toward broader examination of how narrative formation and reputational politics intersect with global sport.

Finally, regional authorship patterns show a gradual diversification of the field. While Europe and North America remain dominant, rising contributions from Asia, Latin America, and Eastern Europe mirror geopolitical shifts in global sport and reinforce observations by Brannagan and Reiche (5), Chadwick and Widdop (9), and Ilevbare (22) regarding the expanding strategic engagement of Gulf and Global South states in international sport.

### 5.2 Implications for global sport governance and political communication

Taken together, the cluster structure and interpretative patterns indicate that sports mega-events (SMEs) function as catalysts for political and cultural transformation. The interaction of soft power, governance practices, infrastructural investment, and narrative management positions SMEs as key arenas where states negotiate identity, legitimacy, and geopolitical influence- consistent with findings by Grix, Dinsmore, and Brannagan (23) and Chadwick and Widdop (9).

The rise of media and communication themes across clusters highlights that sportswashing is increasingly embedded in transnational information flows, where digital platforms mediate both state-driven promotional narratives and critical counter-narratives advanced by journalists, NGOs, and global audiences. This aligns with Boykoff's (2) argument that contemporary sport is a battleground of political meaning-making, wherein debates over human rights, labour, gender, migration, and environmental justice shape public perceptions of global sport governance.

The thematic integration observed in the clusters suggests that sportswashing operates as a cross-cutting mechanism linking economic ambition, infrastructural spectacle, and communicative strategy. SMEs thereby become influential instruments shaping, not merely reflecting, global political dynamics, cultural identity, and international reputation. Understanding these interconnections is crucial for strengthening accountability, transparency, and ethical governance in international sport systems.

At the same time, a more balanced interpretation requires recognizing that not all forms of sport-based image creation should be equated with sportswashing. States, cities, and organizations may use sports events for public diplomacy, tourism promotion, urban development, or international visibility without necessarily seeking to conceal reputational harm. Sportswashing is therefore best understood as a narrower concept within a broader spectrum of image creation and soft power practices, particularly where sport is used to offset criticism related to governance, human rights, corruption, inequality, or authoritarian legitimacy. This distinction is important for avoiding conceptual overextension and for comparing democratic, authoritarian, and hybrid political contexts more carefully.

### 5.3 Sports facilities and Urban development as material mechanisms of image creation and sportswashing

The bibliometric results show a strong intersection between sport-based image creation, sportswashing, and themes related to the urban economy, sports facilities, and stadium development within the red cluster. This indicates that contemporary image-building through sport is increasingly materialized through the built environment. Stadiums, arenas, fan zones, transport corridors, and broader urban redevelopment projects are not only functional components of event hosting but also visible symbols through which states, cities, and organizations communicate modernization, capacity, ambition, and legitimacy. Recent work on sustainability in sports facilities further shows that environmental performance, resource efficiency, and long-term facility management have become important dimensions in evaluating the developmental claims attached to sport infrastructure (24). In reputationally contested cases, these material strategies may also operate as mechanisms of sportswashing.

This interpretation is consistent with scholarship arguing that mega-event hosting and stadium construction function not only as economic projects but also as political acts that spatially manifest state power and legitimacy (3, 8, 11). Sports infrastructure thus represents a tangible dimension of sport-based image creation and, in specific cases, sportswashing, where symbolic nation-branding converges with urban planning, investment, and political communication.

Across these works, large stadiums and associated transport systems are interpreted as architectural markers of modernization and global integration. Such infrastructural spectacles signal state capability and reinforce political legitimacy domestically and internationally. As Giulianotti and Klauser (11) note, these material transformations operate as national spectacles in which space itself becomes a vehicle of political narrative construction. The bibliometric indication that sportswashing and sport-based image creation are closely linked to urban development reflects this recognition: roads, arenas, fan zones, and transport corridors function as instruments through which states articulate ambition, authority, and international visibility.

Recent case studies reinforce this infrastructural turn. Qatar's 2022 FIFA World Cup exemplifies the use of sports infrastructure as a soft-power and image-building instrument. Brannagan and Reiche (5) show that the Lusail Stadium, part of Qatar's broader “megaproject phase” and aligned with similar Gulf developments in Abu Dhabi and Dubai, was designed to project sustainability and modern governance. At the same time, extensive human rights concerns during tournament preparation, documented by Human Rights Watch (2022)^2^, exposed the contradictions in such image-building. In this case, the term sportswashing is analytically appropriate because infrastructure-led image creation was directly entangled with international criticism concerning labour rights, human rights, and reputational management. These mismatches reflect what scholars describe as “dual-use infrastructure”: facilities serving both international spectacle and domestic political consolidation.

Russia's Sochi 2014 Winter Olympics display similar dynamics. With more than USD 50 billion invested in venues and transport systems (25), the event symbolized national resurgence while ultimately leaving behind unused facilities, environmental degradation, and opaque financial governance. These outcomes align with Preuss's (26) emphasis on urban legacy as a critical lens in mega-event research and illustrate how infrastructure can also function as a geopolitical signal, preceding Russia's annexation of Crimea and reinforcing narratives of territorial assertion.

Comparable mechanisms may also appear in democratic or hybrid political contexts, although they should not automatically be described as sportswashing. Hungary's stadium construction boom under Viktor Orbán illustrates how sports infrastructure can be politically instrumentalized for domestic legitimacy. The Pancho Arena and other publicly funded stadiums serve as symbols of national revival and identity politics (BBC Sport, 2024)^3^, echoing Boykoff's (12) argument that sport can amplify nationalist sentiment. However, this case is better understood as domestic image creation and political legitimation through sport infrastructure rather than sportswashing in the narrower international-reputation sense. Its inclusion illustrates the broader conceptual field within which sportswashing is situated.

Taken together, bibliometric patterns and empirical cases suggest that sports infrastructure has become one of the most visible material arenas through which sport-based image creation, and in reputationally contested cases, sportswashing, is enacted. Stadiums and urban redevelopment projects generate quantifiable indicators such as investment, employment, visitor flows, and urban renewal, which allow governments and organizing bodies to legitimize image-building efforts within technocratic and developmental frameworks. Yet these indicators can obscure socio-political inequalities, uneven developmental outcomes, labour concerns, environmental costs, and opaque governance arrangements. This reinforces the paradox highlighted in the literature (3, 8, 11): although infrastructure appears in keyword networks as a marker of development and modernization, scholarship frequently emphasizes its contradictory and sometimes extractive realities.

Overall, infrastructure operates as both an economic mechanism and a political instrument. It attracts capital, tourism, and international visibility while simultaneously signalling progress, stability, and state capacity. Recognizing this dual function is essential for connecting bibliometric trends to the qualitative dynamics that shape global sport governance and the political economies of mega-event hosting.

In summary, the integration of economic, spatial, and political perspectives reveals that infrastructure is not a passive outcome of sport policy but an active agent in the reproduction of power. By embedding sport-based image creation, including sportswashing in reputationally contested cases, within the material fabric of cities, governments, and organizing bodies use the built environment to stabilize political narratives and project soft power. Recognizing this dynamic expands the discussion beyond media and governance frameworks toward a more comprehensive understanding of how architecture, policy, investment, and symbolism intertwine in the global politics of sport.

### 5.4 Conceptual boundaries: image creation, soft power, and sportswashing

The findings also point to the need for clearer conceptual boundaries between image creation, soft power, sport diplomacy, nation branding, and sportswashing. Image creation is the broadest category, referring to the use of sport to construct visibility, attractiveness, credibility, and international recognition. Soft power and sport diplomacy refer to more specific forms of influence and relationship-building through sport. Sportswashing, by contrast, should be understood as a narrower and more normatively charged concept, applicable when sport investment, event hosting, sponsorship, or infrastructure development is used to offset, obscure, or reframe reputational problems in other domains.

Recent conceptual work also emphasizes that sportswashing should be analysed as a complex assemblage of state investment, global sport institutions, sponsorship, media narratives, and reputational strategies rather than as a single, uniform practice (27).

This distinction is particularly important in research that connects sport diplomacy, soft power, and sportswashing within broader foreign-policy strategies (28). The bibliometric results further support this conceptual differentiation, showing that sportswashing is present in the network but does not dominate it. Its lower frequency and concentrated linkages suggest that it functions as an emerging subfield within broader research on sport events, governance, image creation, and political communication. Treating sportswashing as one concept within this broader landscape allows for a more balanced interpretation and avoids reducing all sport-based image strategies to reputational laundering.

Future research should therefore differentiate between general image-building, legitimate public diplomacy, development-oriented event hosting, and cases where sportswashing is analytically justified. Such differentiation would strengthen comparative analysis across democratic, authoritarian, and hybrid regimes and improve the conceptual precision of research on sport events as economic and political instruments.

### 5.5 Future directions

The findings of this bibliometric analysis indicate several priorities for advancing research on sport-based image creation, soft power, governance, and sportswashing. A further priority is conceptual precision. Future research should distinguish more clearly between image creation, soft power, sport diplomacy, nation branding, and sportswashing, rather than treating these terms as interchangeable. Although scholarly interest has expanded, the field remains conceptually dispersed and empirically limited. Three key avenues for future work emerge.

First, there is a need for systematic quantitative research capable of assessing the long-term economic, social, and political consequences of sportswashing practices. Existing studies predominantly examine short-term visibility effects and soft power gains, while evidence on sustained economic outcomes, governance improvements, or policy impacts remains scarce.

Second, ethical and governance dimensions require deeper analytical attention. Future studies should investigate how international sport organizations, event owners, sponsors, and host governments either constrain or facilitate sportswashing through allocation decisions, branding strategies, and partnership frameworks. Strengthening transparency, accountability, and stakeholder oversight will be essential for evaluating legitimacy within global sport governance.

Third, geographical imbalances persist. Most publications originate from Europe, North America, and East Asia, leaving a limited understanding of sportswashing processes in developing, transitional, and politically contested regions- the very contexts where sport is increasingly used for nation-branding, reputation management, and foreign investment strategies. Comparative and cross-regional analyses would help illuminate contextual variation and expand theoretical generalizability.

Overall, future research should integrate economic, political, and communicative perspectives to capture the complex mechanisms through which sport functions simultaneously as a developmental tool and a vehicle of legitimacy. Such an approach will support a more globally representative and analytically robust understanding of sportswashing and its implications for contemporary sport governance.

### 5.6 Limitations

This study has several limitations. First, it relies on bibliographic metadata from Scopus, with WoS used as a cross-check; therefore, publications not indexed in these databases, including some regional journals and grey literature, may be underrepresented. Second, the search strategy was keyword-based and may have excluded studies that discuss sport-based image creation, reputation management, or soft power without using the selected terms. Third, although no language restrictions were applied, the final corpus was dominated by English-language publications, reflecting the indexing profile of Scopus and WoS. Fourth, bibliometric mapping identifies co-occurrence patterns and collaboration structures but does not establish causal relationships between sport events, political legitimacy, economic outcomes, and reputational change. These limitations were mitigated through iterative query refinement, Scopus- WoS cross-checking, keyword cleaning, thesaurus validation, and explicit threshold reporting.

## 6 Conclusion

This article demonstrates that research on sports events as economic and political instruments has evolved into a multidimensional field linking image creation, soft power, sport governance, infrastructure development, and reputational politics. Sportswashing appears not as an isolated or all-encompassing concept, but as a narrower and reputationally contested dimension within wider practices of image creation and political communication through sport. The bibliometric evidence reveals a clear disciplinary shift from event-centred analysis toward broader examinations of governance, legitimacy, political communication, and spatial development.

The economic-governance cluster emerges as a key conceptual core of the field, connecting adjacent domains such as media, tourism, education, corporate responsibility, urban development, and political communication. At the same time, the presence of sportswashing, soft power, and sport diplomacy within the co-word network indicates that symbolic, reputational, and political dimensions are increasingly integrated into research on sport events. This supports the interpretation of sportswashing as an embedded and emerging concept within a broader research landscape rather than as a separate or dominant research stream.

The analysis also highlights the growing geographical diversification of the field, with increasing contributions from Asia, Latin America, and Eastern Europe, broadening methodological approaches and enriching theoretical perspectives. This expansion reflects a maturing and increasingly interdisciplinary research landscape, while also showing that knowledge production remains shaped by regional asymmetries and uneven international collaboration patterns.

More broadly, the findings show that sports mega-events function as catalysts for political, cultural, and spatial transformation. Themes associated with soft power, governance, sport infrastructure, and urban development demonstrate how SMEs operate as arenas in which states, cities, and organizations negotiate legitimacy, identity, visibility, and geopolitical influence. Infrastructure megaprojects, in particular, materialize these political narratives by embedding state ambitions within the built environment.

Taken together, these insights underline that sportswashing should be understood as a specific and reputationally contested dimension within broader practices of sport-based image creation, soft power projection, and political communication. Its significance lies not in replacing wider concepts such as image creation, sport diplomacy, governance, or nation branding, but in highlighting cases where sport events, infrastructure investment, or sponsorship are used to manage, reframe, or offset reputational concerns. Understanding this relationship is essential for analysing how SMEs shape global narratives, influence governance practices, and contribute to international reputation-building strategies. Continued investment in integrative, evidence-based, and comparative research will be crucial for developing a more conceptually precise and accountable understanding of how sport functions not only as a driver of development and visibility but also as a reflection of power relations in the twenty-first century.

## Data Availability

The original contributions presented in the study are included in the article/Supplementary Material, further inquiries can be directed to the corresponding author.

## Footnotes

^1^VOSviewer online bibliometric co-word network map: https://app.vosviewer.com/?json=https%3A%2F%2Fdrive.google.com%2Fuc%3Fid%3D1xqHLEIy2tbm6IopzMia5Di0pT1xE1vSe

^2^Human Rights Watch. (2022). *Qatar: Rights abuses stain FIFA World Cup.* Available at: https://www.hrw.org/news/2022/11/14/qatar-rights-abuses-stain-fifa-world-cup [Accessed December 1, 2025].

^3^BBC Sport. (2024). *Hungary’s Euro 2024 ambitions and Viktor Orban’s politics.* Available at: https://www.bbc.com/sport/football/articles/cekk4zp5rg8o [Accessed June 20, 2024].
